# Comparison of the External Morphology of the Sternal Glands for Hornets in the Genus *Vespa*

**DOI:** 10.3390/biology11020245

**Published:** 2022-02-05

**Authors:** Heather R. Mattila, Gard W. Otis, Johan Billen, Lien T. P. Nguyen, Satoshi Shimano

**Affiliations:** 1 Department of Biological Sciences, Wellesley College, Wellesley, MA 02841, USA; 2 School of Environmental Sciences, University of Guelph, Guelph, ON N1G 2W1, Canada; gotis@uoguelph.ca; 3 Zoological Institute, University of Leuven, Naamsestraat 59, Box 2466, B-3000 Leuven, Belgium; johan.billen@kuleuven.be; 4 Insect Ecology Department, Institute of Ecology and Biological Resources, Vietnam Academy of Science and Technology, Hanoi 10000, Vietnam; phuonglientit@gmail.com; 5 Science Research Center, Hosei University, Fujimi, Tokyo 102-8160, Japan; sim@hosei.ac.jp

**Keywords:** social insects, social wasps, Vespinae, exocrine glands, van der Vecht gland, Richards gland, giant hornets, group predation, pheromone communication, chemical defense

## Abstract

**Simple Summary:**

Hornets are a group of 22 species of highly social wasps in the genus *Vespa.* These conspicuous insects construct large nests that are maintained by one or more queens and their offspring. Unlike other insects, many species of social wasps have glands on the underside of their abdomen called the van der Vecht and Richards glands. Documented uses for these glands by workers of some hornet species include producing pheromones for nestmate recruitment during group attacks on prey and chemical defense of nests against ants. We confirmed that these glands were present in nine hornet species. To examine differences among species in gland morphology, we used scanning electron microscopy to image the external surface of worker bodies around the glands. Accounting for differences in body size, we found that giant hornets and their close relatives had comparatively larger glands than the other species, some of which had glands that were approximately half as large. Differences among species are best explained by how hornets hunt and not by the likelihood that their nesting habitat increases exposure to ant predation. However, more information about how hornet workers use their sternal glands is necessary to permit further interpretation of their comparative gland morphology.

**Abstract:**

Many social wasps in the speciose subfamilies Polistinae and Vespinae have two sternal glands—the van der Vecht gland and the Richards gland—that are not found in other insects. The presence of these glands has been confirmed in only 6 of 22 hornet species (genus *Vespa*) and images of their fine structure have not been produced. Here; we characterize the external morphology associated with both glands for workers of nine *Vespa* species using scanning electron microscopy. All hornets had similar gland configurations; although gland-associated external features differed among species. Scaled for size, glands were equivalently sized for the giant hornets (*V. mandarinia* and *V. soror*) and their closest phylogenetic relatives (*V. tropica* and *V. ducalis*). Relative size of gland-associated structures was reduced by half for *V. simillima*; *V. velutina*; and *V. affinis* workers. The remaining species (*V. crabro* and *V. analis*) had intermediately sized features. Differences among species in external gland structure were best explained by selective pressures related to predatory behavior, rather than defense of nests against ants. However, a lack of information about how *Vespa* workers use their van der Vecht and Richards glands limits a comparative interpretation of the function of their external gland morphology.

## 1. Introduction

**Figure 1:**
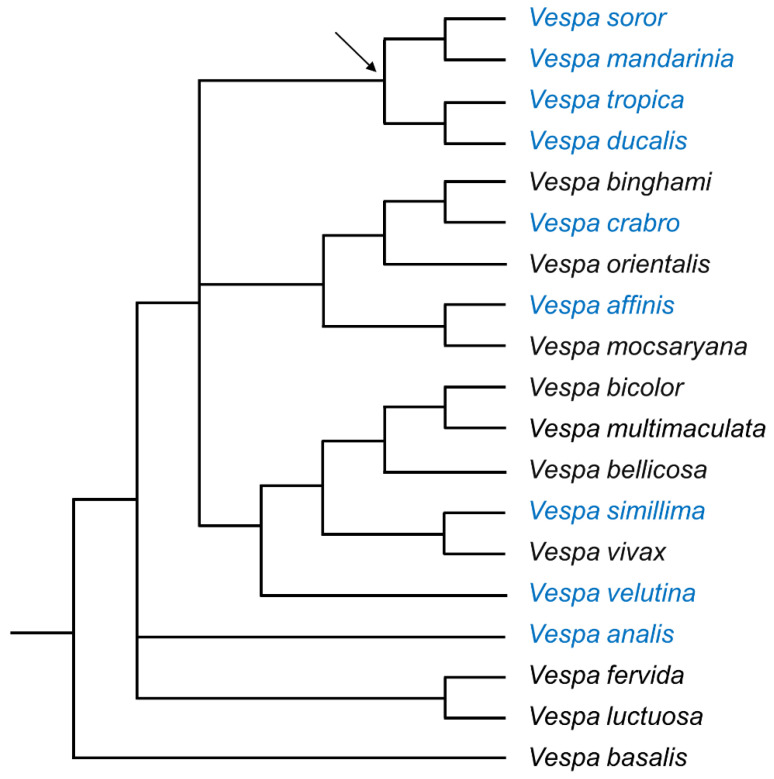
Phylogeny of the genus *Vespa* (19 of 22 species). Relationships are supported by a combined analysis of morphological characteristics, wing-venation landmarks, and molecular data, modified after Perrard et al.’s Figure 8 [4]. The nine species examined in this study are indicated by blue text; the arrow indicates the root of the ‘*tropica* group’ [4,14].

*Vespa* Linnaeus, 1758 (Hymenoptera: Vespidae: Vespinae) is a biologically diverse genus of 22 species of hornets that share many morphological, behavioral, and ecological traits (Figure 1) [1,2,3,4]. These conspicuous insects are mostly restricted to Asia, although two species have natural distributions that extend to parts of Europe, North Africa, and the Middle East [5]. All hornets form eusocial colonies with morphologically differentiated castes of reproductives and workers [1,6]. In general, overwintered foundresses of both temperate and tropical species establish nests in the spring or early summer and rear their first generation of workers. Thereafter, workers assume most duties related to nest construction, foraging, and brood care, including rearing new reproductives toward the end of the annual cycle [1,2,6]. The nests, initiated by queens either above or below ground depending on the species, are expanded by the workers. Hornets forage from plants and fungi [1] and can even be important pollinators [7,8], but they are better known as voracious predators of arthropod prey that adults feed to their larvae [1,6,9]. Most hornets hunt solitarily, although giant hornets are well known for hunting in groups [1,2,6,9,10,11,12,13].

The collective behaviors of hornets and other social wasps, most of which are in the subfamilies Vespinae (to which *Vespa* belongs) and Polistinae [15,16], are supported in part by exocrine glands and their secretions (reviewed by [17]). Of particular interest are the social functions and morphological features of two sternal glands that, unlike other exocrine glands, are found in some species of vespids only (reviewed by [17,18]). They are the van der Vecht gland, located on the sixth metasomal (=terminal) sternite, and the Richards gland, located on the fifth metasomal (=penultimate) sternite. When present, these glands tend to be well developed and clearly discernible, and are often associated with external cuticular modifications that are presumably related to how gland products are used [17,18]. Following the standard classification by Noirot and Quennedey [19], there are two categories of insect glands: class 1 glands are formed by a single layer of epithelial cells and class 3 glands are formed by bicellular units, each unit comprised of a secretory cell and a duct cell (an initial classification also included class 2, which was later recognized as homologous to oenocytes [20,21]). Both the van der Vecht and Richards glands are class 3 glands [18,19], the ducts of which open to external pores at the anterior margin of each sternite. Pores of the van der Vecht gland occur in two lateral clusters, while external configuration of the Richards gland is more variable across vespids [22,23,24,25,26,27,28].

These two sternal glands have been examined extensively among the polistines, primarily because both gland occurrence and social behaviors vary broadly within this speciose subfamily (reviewed by [17,18]). Gland presence, morphology, and function have been reported to a lesser extent within the vespines (reviewed by [17,26,28]). Across species examined to date in both subfamilies, females have a van der Vecht gland if they found nests independently, but the gland is absent in most species that found nests as swarms [17,18,22,23,25,28,29,30,31,32]. An important function of gland secretions is to protect unguarded nests from ant predation. Gland products repel ants and foundresses rub their glands on nest petioles in the species that have been studied [31,33,34,35,36,37,38,39,40,41,42,43]. Additionally, secretions from the van der Vecht gland have been found to play a role in queen dominance in *Polistes* [44,45] and nestmate recruitment in the giant hornets *V. mandarinia* and *V. soror* [11,32]. It has been more difficult to identify a general function that correlates well with gland presence for the Richards gland [26]. Depending on the species, its secretions are involved in swarm guidance [25,46,47], ant repellency [31,41], and attraction between reproductives [48]. Like the van der Vecht gland, the Richards gland occurs widely across the polistines and vespines, but is absent or poorly defined in many species [17,18,22,23,24,25,26,27,28,29,49,50,51].

This study presents a comparison of the external morphology associated with the sternal glands of 9 of the 22 hornet species in the genus *Vespa*. At present, the structure and function of hornets’ glands have not been extensively explored, even though they likely facilitate their ecological success as social insects and their impact as pests to humans and predators of economically important pollinators [52,53,54,55,56,57,58,59]. Published information about sternal gland structure has been limited to drawings of the van der Vecht and Richards glands for *V. crabro*, *V. tropica*, and *V. affinis* [29,31], and of the van der Vecht gland only for *V. analis* [30]. Recently, we completed a microscopic analysis of the internal and external fine structure of both glands for the giant hornet *V. soror* [32]. In the present study, we extend that work to an examination via scanning electron microscopy (SEM) of the external features associated with the van der Vecht and Richards glands of *V. affinis*, *V. analis*, *V. crabro*, *V. ducalis*, *V. mandarinia*, *V. simillima*, *V. tropica*, and *V. velutina*. These hornets share genus-related commonalities, but are also characterized by species-specific traits that can be tied to sternal gland function. For instance, the giant hornets *V. soror* and *V. mandarinia* recruit nestmates to prey colonies using secretions of the van der Vecht and possibly other gastral exocrine glands [11,32]. Other species-specific behaviors may also benefit from social communication facilitated by sternal glands. For instance, these species differ in their distributions and nesting habits [1,2], and thus their risk of exposure to ant predation [9,60,61]. Additionally, many *V. simillima*, *V. crabro*, and *V. velutina* colonies undergo nest relocation during their annual cycles [1,2,6,9,62,63,64,65,66,67], for which orientation mechanisms are unknown.

Our goal is to characterize the external fine structure of the van der Vecht and Richards glands for workers of nine species within the genus *Vespa* (Figure 1), and then consider similarities and differences in gland-associated morphology in the context of the hornets’ natural histories. Notably, seven of these species have been detected or become established after accidental introduction to continents outside of their natural ranges [5,68,69], highlighting the need for greater understanding of basic aspects of their social biology. Our study shows that hornet workers of all the species we examined have well-developed sternal glands, but that species vary in the expression of gland-associated features. We provide a detailed exploration of the external sternal gland structure within the genus *Vespa*, and consider the selective pressures that have shaped these exocrine glands as they support group living within this important clade of predatory social wasps.

## 2. Materials and Methods

### 2.1. Worker Specimens

Specimens of *Vespa* workers were acquired from several sources for imaging via SEM; Table 1 summarizes their collection information. None of the hornets we imaged are considered endangered in the countries in which the specimens originated, so special collection permits were not required for this study. We studied a total of nine species, with 1–5 specimens per species. Generally, variation within species in gland presence has not been observed previously [25,26,28]; our recent examination of the sternal glands of 25 *V. soror* specimens supports this expectation [32].

### 2.2. SEM Imaging

The fifth and sixth metasomal sternites were carefully removed from the gasters of specimens of each species. Pinned specimens were either relaxed in a humidifier or soaked in 80% ethanol prior to dissection. Specimens were dissected, dried if needed, and sputter coated prior to imaging with a scanning electron microscope. *V. affinis*, *V. analis*, *V. mandarinia*, *V. simillima*, *V. soror* (specimens collected in 2020), and *V. velutina* were imaged via a JEOL–JCM-6000PLUS microscope (JEOL Ltd., Tokyo, Japan). *V. crabro* workers from Belgium were imaged with a JEOL-JSM-6360 microscope. Images of the remaining specimens were taken with an FEI Quanta FEG 250 (FEI Company, Hillsboro, OR, USA). In all cases, external structures of sternites were imaged across a range of magnifications to allow the size of gland-associated structures to be estimated (see next section).

### 2.3. Image Analysis

Features of the fifth and sixth metasomal sternites for the nine *Vespa* species were quantified using ImageJ image analysis software (National Institutes of Health, Bethesda, MD, USA). To get accurate estimates of the size of sternal features, our general approach was to measure each feature in multiple images per specimen across a range of angles and magnifications. Within a single image, length and area measurements were calibrated using the image’s embedded scale (ImageJ: set scale and measure functions). Each area estimate for an image was an average based on triplicate measures taken from that image. All size estimates for a single feature made across multiple images of a specimen were averaged and, if we had more than one specimen per species, average values per specimen were averaged across specimens within a species. Using this approach, one to eight images were used to estimate the size of each sternal feature per species. For some of the larger hornets, composite sternal images were created using multiple images from the same specimen.

We quantified several sternite features, as indicated in Figure S1. The area of cuticle covered with pore openings, from which glandular secretions are emitted, was estimated for each sternite (Richards gland: single anterior band of pores; van der Vecht gland: both lateral pore clusters combined). We also determined the area on the sixth sternite of the hyaline region, which contains the sternal brush between paired pore clusters (demarcated posteriorly by a depression in the cuticle). For both sternites, we calculated the percentage of the sternite’s area (from its anterior margin to the posterior margin of the smooth cuticle; see Figure S1) that was covered by pores. We made the same comparison for the area of the hyaline region relative to the area of the sixth sternite. Mean pore diameter for each gland was estimated from multiple high-magnification images per species (ImageJ: straight line tool; measured range 68–110 pores per gland per species, pooled across specimens as images permitted).

We also estimated the number of external pores per gland per species, which reflects the number of internal bicellular glandular units per gland [18,19,20]. We confirmed this gland classification in *V. soror* [32] and assumed that glandular cells were similarly structured throughout *Vespa*, as other authors have reported for polistine species (e.g., [25]). For the van der Vecht gland, we estimated total number of pore openings per specimen by counting pores in images that fully captured each gland cluster (ImageJ: multi-point tool and grid function). There was minimal overlap between the pore clusters and sternal brush, and pores among setae were counted when visible. In some cases, pores adjacent to the sternal brush could not be counted because the cluster was partially obscured (e.g., by a folded intersegmental membrane or debris). However, we had clear views of at least one cluster per specimen, so in these cases we counted pores in the unobstructed cluster and then doubled that count to estimate total number of pore openings per specimen. For the Richards gland, we calculated the total number of pore openings per specimen by multiplying gland area by average pore density (pores/μm^2^); the latter was estimated from a range of two to nine high-magnification images taken across each specimen’s band of pores.

Finally, for the sixth sternite, we assessed the length of the setae of the sternal brush for each specimen relative to sternite size by dividing the hyaline region into quarters from side to side, then measuring the maximum length from the anterior margin of the sternite to the tip of the longest setae in each quadrant (ImageJ: straight line tool; see Figure S1). These values were averaged and compared to the length of the sternite at the midline for each specimen (to the posterior margin of the smooth cuticle). These calculations were performed for multiple images per specimen as available, and then averaged for each species across available specimens.

### 2.4. Statistics

We had only one specimen for some species, so statistical analyses were limited to avoid pseudoreplication. The only exception was for pore diameter, which was treated as an experimental unit by sampling pores across different images within a species, as image availability and specimen condition permitted. Pore diameter was compared among species using one-way ANOVAs for each gland; data were log transformed to improve normality and means were separated using Tukey HSD tests. Trait values were correlated using Spearman rank-order correlations. Significance for all tests was set at α = 0.05. Tests were conducted using SAS (version 9.3; SAS Institute, Cary, NC, USA).

## 3. Results

All *Vespa* specimens that we examined had obvious van der Vecht and Richards glands, although there were differences among species in the details of their external features. Below, we highlight these patterns.

### 3.1. Structure of the van der Vecht Gland among Vespa Species

**Figure 2:**
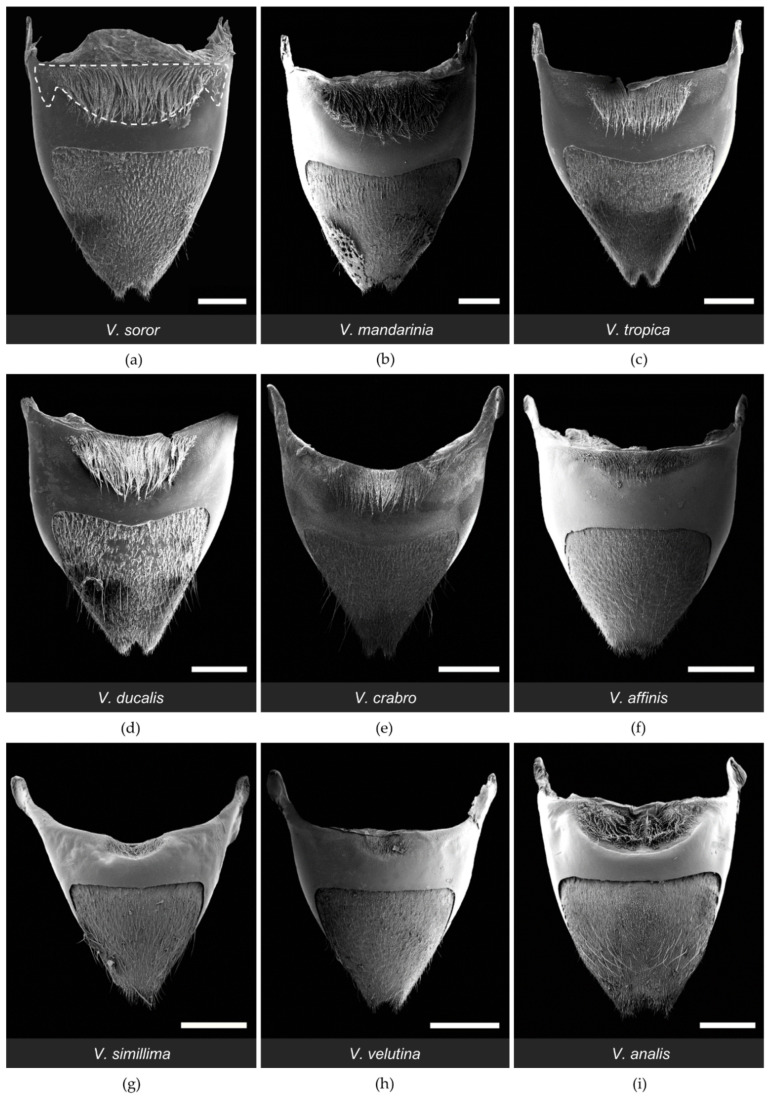
SEM images of the sixth metasomal sternite and van der Vecht gland of nine *Vespa* species (**a–i**). Anterior is at the top. Species are presented in the order in which they appear in the phylogeny depicted in Figure 1. Lateral pore clusters and the hyaline region with the sternal brush are outlined by a dashed white line in (**a**). Bars indicate 1 mm for scale.

**Figure 3:**
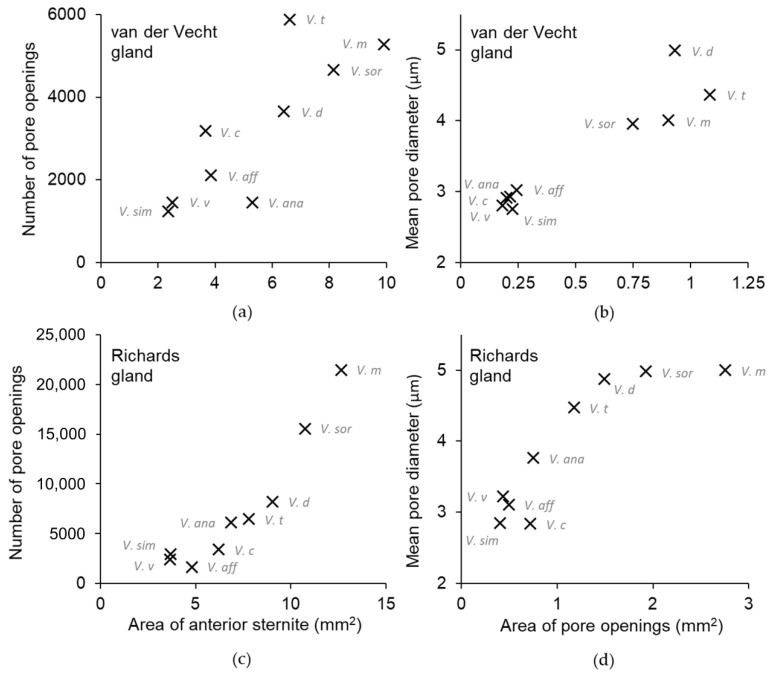
Significant correlations across species between features of the sternal glands. (**a**) Van der Vecht gland size (number of pore openings) increased as the size of the sixth metasomal sternite increased. (**b**) Mean pore diameter increased as van der Vecht gland size (area of pore openings) increased. (**c**,**d**) The same relationships are provided for the Richards gland on the fifth metasomal sternite. All estimates were pooled across images of specimens within species (species abbreviations are in gray).

**Figure 4:**
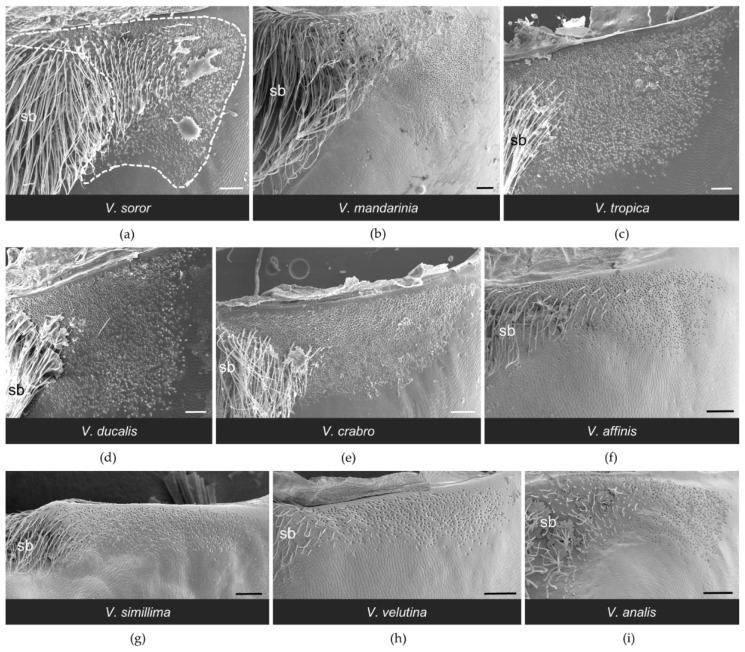
SEM images of the lateral cluster of pore openings of the van der Vecht gland on the sixth metasomal sternite of nine *Vespa* species (**a–i**). Anterior is at the top. Species are presented in the order in which they appear in the phylogeny depicted in Figure 1. The boundary of a lateral pore cluster is outlined by a white dashed line in (**a**). Bars indicate 100 μm for scale; sb = sternal brush.

All workers had paired clusters of van der Vecht gland pores on either side of a medial sternal brush (Figure 2). However, the size of the aforementioned gland features varied greatly across species (Table 2). There was a five-fold difference between species with the smallest and largest glands based on number of pore openings per specimen (range 1200–5900 pores per gland across species; Table 2), which directly reflected number of glandular units internally. Pore number was strongly and positively associated with differences in sternite size among the nine focal species, with gland size increasing with hornet size (Figure 3a; Spearman correlation: r = 0.83, *p* = 0.005). In specimens of all species, pore openings at the inner margins of the pore clusters overlapped slightly with short setae at the lateral edges of the sternal brush, but were absent among longer, more central setae (Figure 4).

**Figure 5:**
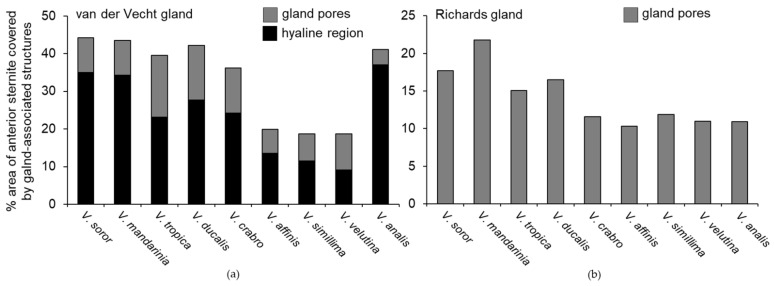
Gland area relative to sternite size across *Vespa* species. Species are presented in the order in which they appear in the phylogeny depicted in Figure 1. (**a**) Percentage of the total surface area of the anterior cuticle of the sixth metasomal sternite that was covered by gland-associated structures of the van der Vecht gland, which includes area of the paired pore clusters added to the area of the hyaline region with the sternal brush. (**b**) Percentage of the anterior of the fifth metasomal sternite that was covered by the band of pores of the Richards gland.

One of the most obvious differences among species was the extent of development of the sternal brush (Figure 2). In absolute terms, the giant hornets (*V. soror* and *V. mandarinia*) had the largest sternal brushes, with long setae that extended beyond the boundaries of the large hyaline regions from which they originated. However, giant hornets also had the largest sternites (Table 2). When scaled to account for sternite size, six species had a similar proportion (~40%) of the anterior part of their sternite occupied by gland-associated structures (i.e., pores and the hyaline region; Figure 2a–e,i and Figure 5a). Almost all of these species (i.e., *V. soror*, *V. mandarinia*, *V. tropica*, *V. ducalis*, *V. crabro*) also had long setae that extended beyond the cuticular depression that marked the posterior margin of the hyaline region (Figure 2a–e, Table 2). The exception was *V. analis* (Figure 2i, Table 2). In contrast, three species—*V. affinis*, *V. simillima*, and *V. velutina*—had modest external structures associated with their van der Vecht glands that covered less than 20% of the anterior part of their sternites (Figure 2f–h and Figure 5a; Table 2). For these species, the sternal brush was relatively small, with shorter and sparser setae compared to the more elaborate sternal brushes of the other species. All species had a depression that held the sternal brush, which likely creates a reservoir for gland secretions, as both internal and external ultrastructure has suggested for *V. soror* [32], although storage capacity would be relatively reduced by the small size of the hyaline region in these latter three species (Figure 2f–h). 

**Figure 6:**
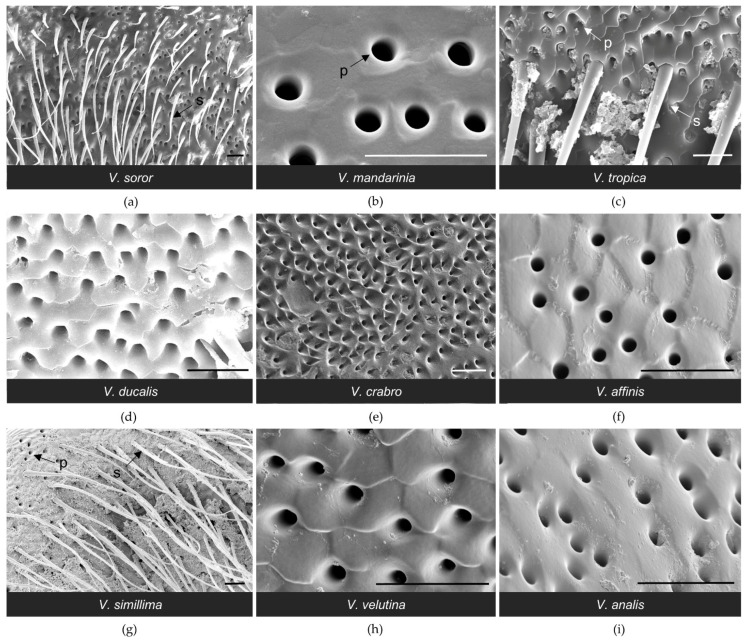
High magnification SEM images of the van der Vecht gland showing differences in cuti-cular texture across species. Anterior is at the top. Species are presented in the order in which they appear in the phylogeny depicted in Figure 1. The region where pores overlap with setae at the margin of the sternal brush is shown in (**a**,**c**,**g**). Regions of pore clusters without setae are shown in (**b**,**d**–**f**,**h**,**i**). Bars indicate 20 μm for scale; s = seta of sternal brush; p = pore opening.

At a fine scale, there was variability both across species and within specimens of the same species in the texture of the cuticle around pore openings of the van der Vecht gland; Figure 4 and Figure 6 highlight some of these differences. The cuticular surface of *V. tropica* and *V. ducalis* had scale-like projections surrounding anterior pore openings, especially approaching the midline (e.g., Figure 6c,d). Less sculptured, wave-like topography was observed for *V. affinis*, *V. simillima*, *V. velutina*, and *V. analis*, with some pores opening into shallow depressions (Figure 6f–i). The majority of species had some or most pores surrounded by smooth cuticle (e.g., Figure 6a,b), with the exception of *V. crabro*, for which most pores opened into the bases of sharply articulated pits (Figure 4e and Figure 6e). The diameter of pore openings differed among species (Table 2; one-way ANOVA: F_8,1012_ = 226.8, *p* < 0.0001) and correlated strongly with gland area (Figure 3b; Spearman correlation: r = 0.88, *p* = 0.002), dividing broadly into species with large pore openings (the giant hornets *V. soror* and *V. mandarinia* and their close relatives *V. tropica* and *V. ducalis*, i.e., the *tropica* taxonomic group [3,4,14]) and species with relatively smaller pore openings (*V. crabro, V. affinis, V. simillima, V. velutina*, and *V. analis*).

### 3.2. Structure of the Richards Gland among Vespa Species

**Figure 7:**
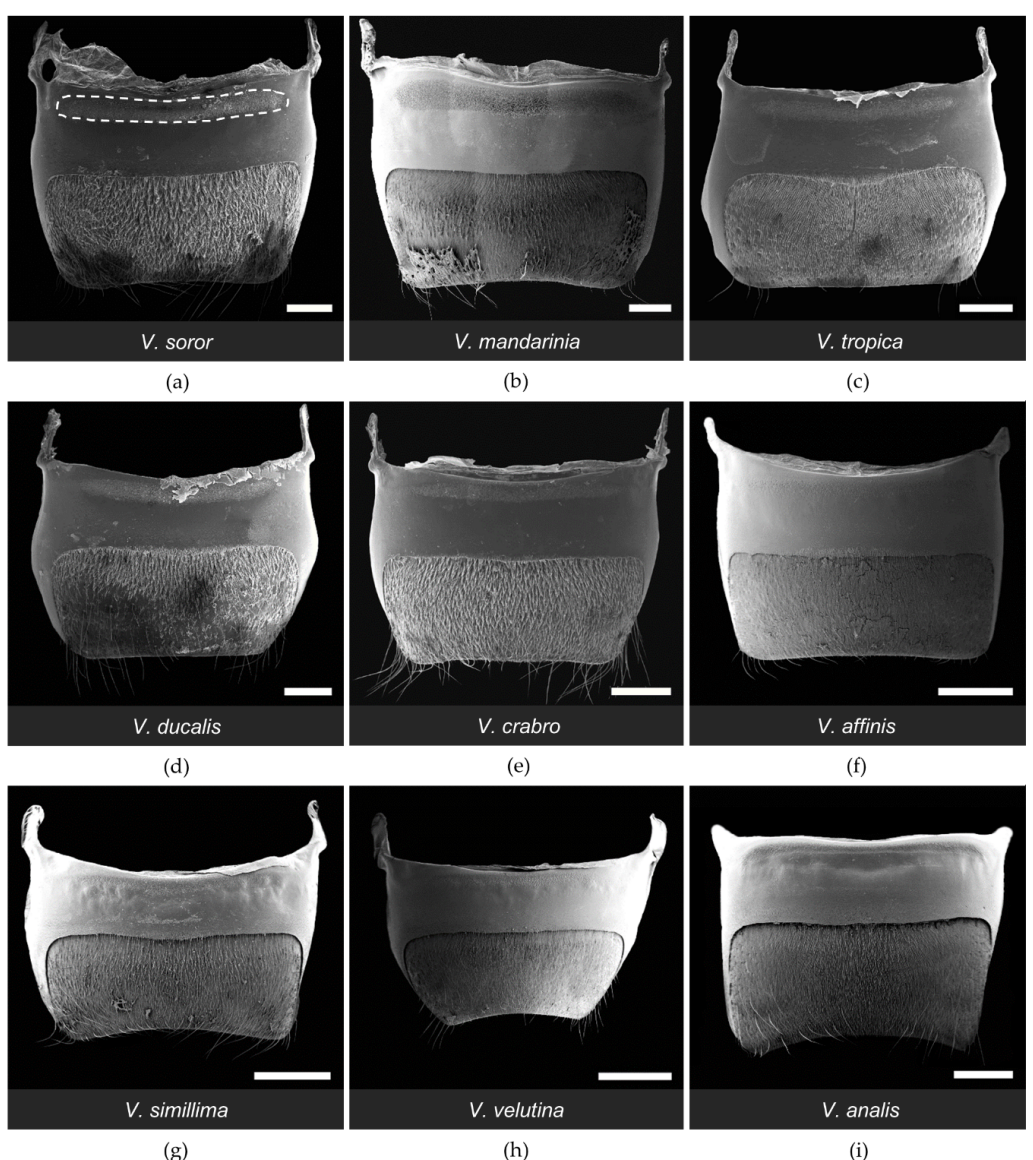
SEM images of the fifth metasomal sternite and the Richards gland of nine *Vespa* species (**a**–**i**). Anterior is at the top. Species are presented in the order in which they appear in the phylogeny depicted in Figure 1. The anterior band of pores of the Richards gland is outlined by a dashed white line in (**a**). Composite images (made from images of the same specimen) are shown in (**b**,**i**). Bars indicate 1 mm for scale.

**Figure 8:**
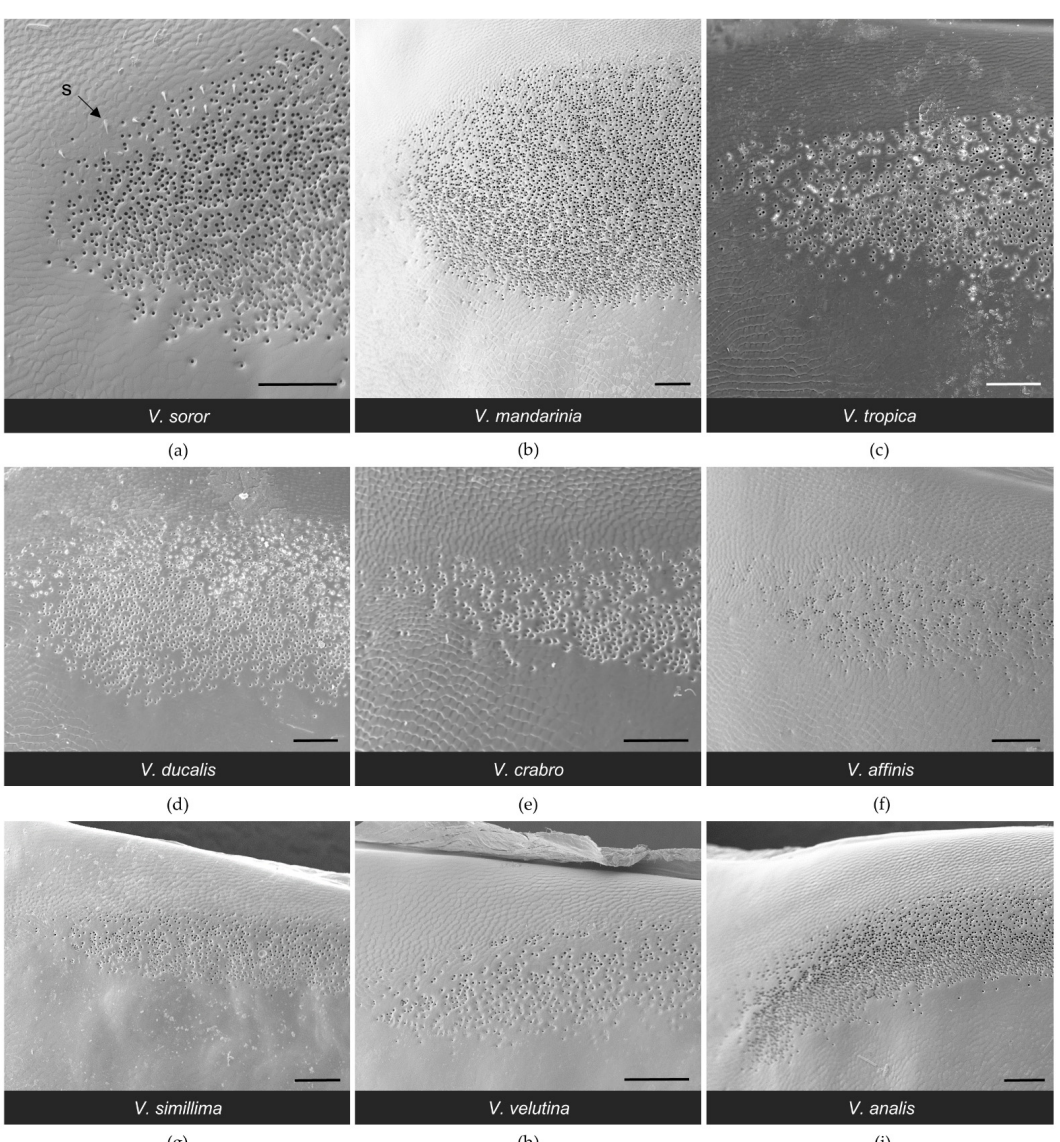
SEM images of nine *Vespa* species (**a**–**i**) showing the lateral margin of the band of pores of the Richards gland. Anterior is at the top. Species are presented in the order in which they appear in the phylogeny depicted in Figure 1. Bars indicate 100 μm for scale; s = seta.

**Figure 9:**
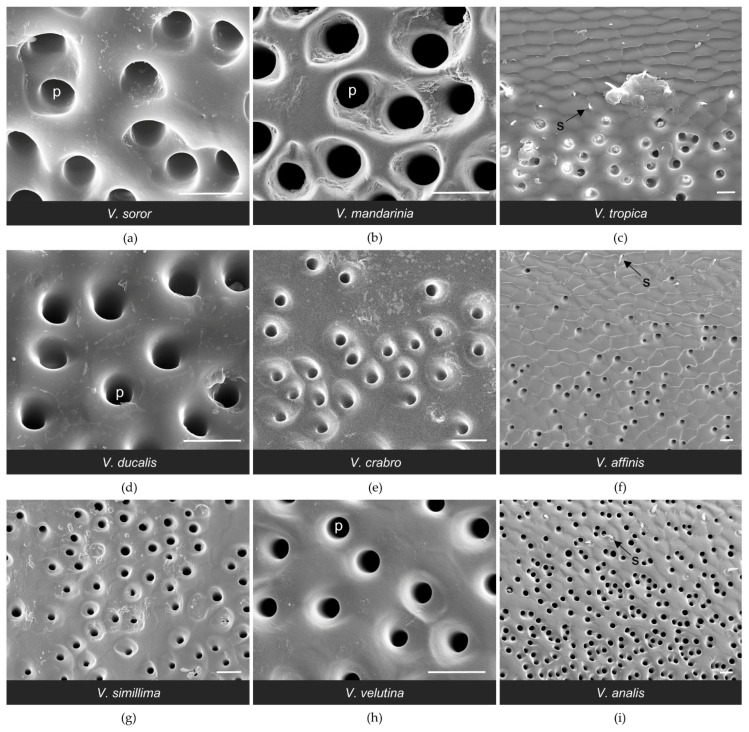
SEM images of nine *Vespa* species (**a**–**i**) showing high-resolution detail for pore openings of the Richards gland. Anterior is at the top. Species are presented in the order in which they appear in the phylogeny depicted in Figure 1. Bars indicate 10 μm for scale; p = pore, s = seta.

The general configuration of the Richards gland was similar among *Vespa* species; all workers had a well-defined region of pores that opened to the cuticle in a continuous band along the anterior margin of the fifth metasomal sternite (Figure 7 and Figure 8). Other than pore openings, this region was relatively featureless, with few cuticular modifications among the specimens that were examined (Figure 7, Figure 8 and Figure 9). All workers had pore openings that were on the same plane as the surrounding cuticle (Figure 9c,d,f), and these pores tended to be located at the anterior edge of the band. However, depending on the species, this transitioned toward the posterior edge of the band to either individual openings that sat at the base of a cuticular depression (Figure 9e,g,h) or pores that clustered in shared depressions (Figure 9a,b,i) that may serve as mini-reservoirs into which secretions accumulate. Figure 9 highlights differences among species in the nature of these depressions, where they occurred. All workers had short, bristly setae that were thinly distributed at the anterior margin of the band of pores (e.g., Figure 8a and Figure 9c,f,i).

The biggest difference among species was the size of their Richards glands, which was reflected in a range from only 1600 pores for *V. affinis* to 15,000 to 21,000 pores for the giant hornets *V. soror* and *V. mandarinia*, respectively (Table 3). Differences in pore number resulted from some key differences among species. Firstly, pore number increased strongly with sternite size (Table 3; Figure 3c; Spearman correlation: r = 0.95, *p* < 0.0001), so larger species tended to have larger Richards glands. Secondly, although the proportion of the fifth sternite that the Richards gland occupied was not starkly different among species, the giant hornets and their close relatives (*V. tropica* and *V. ducalis*) had >15% of their fifth sternites covered with pores, whereas the remaining species had ~10% coverage of their sternites by pores (Figure 5b). Moreover, some species had more sparsely distributed pores within gland boundaries. For instance, there were noticeable differences in pore density for *V. affinis* and *V. velutina* (Figure 8f,h) compared to the giant hornets, as well as *V. tropica*, and *V. ducalis* (Figure 8a–d). Lastly, we found that pore size increased across species as gland area increased (Figure 3d; Spearman correlation: r = 0.88, *p* = 0.002). Pore diameter was largest in the giant hornets and their close relatives (Figure 3d; Table 3; one-way ANOVA: F_8,1043_ = 392.4, *p* < 0.0001).

## 4. Discussion

All workers of the nine *Vespa* species that we examined in this study showed prominent external evidence of sternal glands on their fifth and sixth metasomal (=penultimate and terminal) sternites. While information about how *Vespa* workers use these glands is limited (as discussed below), their substantial size suggests that their function is important. The basic features of these glands were similar across species and in line with configurations reported for both glands in surveys of other vespids [17,18,22,23,24,25,26,27,28,29,30]. Externally, the van der Vecht gland in all *Vespa* species consisted of paired clusters of pore openings on either side of a medial sternal brush at the anterior of the sixth metasomal sternite. The Richards gland always presented as an uninterrupted band of pore openings along the anterior margin of the fifth metasomal sternite. We did not have live specimens or specimens that were preserved in a way that permitted an examination of the internal cell structure of either gland (in contrast to our detailed study of *V. soror* [32]). However, external pores are indirect evidence of class 3 gland structure, with each pore leading to a duct cell that provides passage for the products of the single secretory cell with which it interfaces [19,25,70]. Our recent histological examination of *V. soror* workers confirmed the association between external pores and these bicellular glandular units [32]. It is possible that class 1 glandular cells were also present—they have been noted in other vespids [23,25,27]—but these would not be detectable from external SEM images only. Our work supports statements about the ubiquity of the van der Vecht gland throughout the Vespinae [2,31] and expectations that the same is true for the Richards gland within *Vespa* based on previous examination of two species (*V. crabro* and *V. orientalis* [22,26]. In fact, until recently, details about the presence of one or both glands had been available for five *Vespa* species only, with drawings of the position of external features published for four of those species [22,29,30,31]. Our work extends our understanding of the external morphology of these glands for almost half of the members of the genus (this study, [32]).

There were notable trends in the external features of both sternal glands across species. In general, larger hornets had larger glands, represented by more pore openings, as well as larger gland-related features, such as wider pore diameter and increased size of the hyaline region and sternal brush. Size matters, as it has widely been assumed that larger gland features are indicative of increased functionality when gland morphology is compared across castes or species [23,71,72,73,74,75]. However, body parts may or may not scale proportionally across species when workers differ in size. For instance, the giant hornets *V. mandarinia* and *V. soror* had the largest gland features relative to most other *Vespa* species, but these two species are also the largest hornets. When scaled to sternite size, ~40% of the anterior sternite of many species, including the giant hornets, was covered by the elaborate external features of the van der Vecht gland. However, three species (*V. affinis*, *V. simillima*, and *V. velutina*) had markedly smaller gland features that covered only half that area (i.e., ~20% of the anterior sternite). Differences among species were not as stark for the Richards gland: a modest increase in relative gland coverage was limited to only the giant hornets and their phylogenetically close relatives, *V. tropica* and *V. ducalis*. This same clade of hornets, plus *V. crabro*, also had similarly elaborated sternal brushes associated with the van der Vecht gland, with dense setae that extended beyond the posterior margin of the hyaline region. In contrast, the relatively poorly developed gland-associated features of *V. affinis*, *V. simillima*, and *V. velutina* were matched by comparatively less numerous, smaller, and thinly distributed pores of the Richards gland. *V. crabro* and *V. analis* were generally intermediate, with a mix of both relatively prominent and reduced features.

These broad trends align in many ways with our current understanding of the phylogeny for *Vespa* (Figure 1) [4]. The ‘*tropica* group’ (giant hornets and their closest relatives) is a consistently resolved clade based on both molecular data and morphological (non-glandular) traits of both males and females (Figure 1, [3,4,14]). When gland traits were compared across species in our study, workers from this lineage—*V. soror*, *V. mandarinia*, *V. tropica*, and *V. ducalis*—regularly grouped together based on their relatively large sternal glands (e.g., pore number, area, and diameter; size of hyaline region and sternal brush). At the other end of the spectrum, the relatively undersized sternal gland features that united *V. velutina* and *V. simillima* mirror their repeatedly resolved, close phylogenetic relationship (Figure 1) [3,4,14,64]. The remaining species in our study—*V. crabro, V. affinis*, and *V. analis*—shifted in their alignment with these two previously mentioned groups of hornets depending on which gland features were considered. For example, *V. analis* had a large hyaline area, but short setae in its sternal brush and a small number of pores for its van der Vecht gland, uniting it generally but not uniformly with the other species with relatively small van der Vecht glands. However, the features of *V. analis*’s Richards gland approached the relatively large size of the specimens in the *tropica* group. Interestingly, this lack of clear fit echoes the varying position of *V. analis* in the *Vespa* phylogeny as the genus has been revised over time, as well as its current placement in a monotypic clade (Figure 1) [3,4,14].

A natural question emerges from our comparative approach: do interspecific differences in external gland features reflect differences in species-specific functionality? There are two documented uses of the sternal glands within *Vespa.* The first is the use of the van der Vecht gland by the giant hornets *V. mandarinia* and *V. soror* for nestmate recruitment during group attacks on social insect prey [11,32]. Secondly, there is an association across the Vespidae broadly between the presence of the van der Vecht gland and nest founding by independent foundresses [28], as well as an ability of secretions from foundresses and workers to repel ants, demonstrated mostly by studies of polistines [33,34,35,36,37,38,39,40,43]. In *Vespa*, Matsuura ([76,77] in [42]) briefly noted that foundresses of aerial-nesting species rub their gasters on nest petioles when colonies are workerless and nest envelopes are incomplete. However, subsequent observations suggest that this protection is employed beyond these specific circumstances. Makino [42] observed a *V. analis* foundress rubbing her gaster on the pedicel of a nest after the nest envelope was completed. Furthermore, Martin [31] demonstrated ant repellency from the secretions of both the van der Vecht and Richards glands of workers and queens for species with different nesting sites: *V. affinis*, which nests aerially in open spaces [2,78], and *V. tropica*, which nests above or below ground in enclosed cavities [1,2,9,65,79].

These observations suggest that secretions from either gland could potentially be used by *Vespa* workers for nest defense, and that repellency is conceivably helpful beyond the pre-emergence period (when workers are present) and under diverse nesting scenarios. In contrast to Matsuura’s observations of foundresses, we did not find strong evidence in workers that an aerial nesting habit was linked to comparatively more developed external sternal gland morphology. *V. affinis*, *V. analis*, and *V. velutina* (to a lesser degree) are reliable aerial nesters [1,2,9,31,64,67,78,80], yet gland structures for workers of these species did not suggest increased functionality relative to other species (i.e., their glands were comparatively small, particularly pore number for the van der Vecht gland). In contrast, the four species in the *tropica* group had universally well-developed sternal gland features, but all of them construct nests underground (although above ground occasionally for the giant hornets and more commonly for *V. tropica* and *V. ducalis*, [1,2,9,12,65,78,79,81,82,83]). Ant predation pressure is expected to be greater at ground level compared to above ground in vegetation [60], which could contribute to selection for well-developed sternal glands in this lineage. However, a more important selective pressure for this group is probably the foraging behavior of workers (see below) because the remaining species—*V. crabro* and *V. simillima*—also regularly nest underground [1,2,9,64,78], but do not show clear affiliation with the oversized gland morphology of the *tropica* group. In general, how much hornet species vary in the need for anti-ant defenses after the founding stage is unclear, as is the nature of those defenses. Large workers may easily deflect ants from nests through physical means alone. A chemical defense could be important for species with relatively small colonies, like those of *V. ducalis*, which has a maximum mean of 15 workers present at maturity in Japan, and are similarly small in China and Taiwan [79]. Furthermore, lack of reports of worker participation in ant defense may be a result of the inherent difficulty of making these observations in enveloped or underground nests, leading to conclusions that only queens of aerial, not-yet-enveloped nests use sternal glands for this purpose [42,76,77].

If a function of either sternal gland in *Vespa* workers is to secrete ant-defense chemicals, it is possible that, rather than nesting habit, geographic location may exert greater influence on gland features. Predation pressure from ants is considered a major selective force that drives nesting biology in social wasps [84], and this pressure increases as latitude decreases in both the Americas and Asia [60,61]. In Asian *Polistes*, observed increases in ant predation with decreasing latitude are paired with a latitudinal gradient in gastral rubbing behavior by foundresses [61]. How these observations extend to *Vespa* workers remains unclear, but if sternal glands contribute to ant defense (as in [31]), we can make some predictions. For example, *V. crabro* and *V. simillima* workers, which are restricted to temperate zones, should have reduced gland structures compared to subtropical or tropical *Vespa* species such as *V. soror*, *V. tropica*, *V. ducalis*, and *V. affinis*. However, this was not always the case. Of the giant hornets, the more southerly *V. soror* should have relatively larger glands than *V. mandarinia* that originate from more northern locations, which was also not the case. Interestingly, intraspecific trends for specimens from geographically widespread locations were suggestive. Gland features of the *V. mandarinia* worker from Taiwan were appreciably larger than those of the specimen from Japan, as were the features of the *V. velutina* workers from Taiwan and Vietnam compared to the specimens from France, where they have been recently introduced from a more northerly region in China [85] (Table S1). More in-depth sampling is necessary to explore the intriguing possibility of intraspecific differences in gland features along latitudinal gradients.

Based on the information that is available about these nine *Vespa* species, hunting behavior better explains comparative gland morphology than defense of nests against ants. A well-documented use of the van der Vecht gland within *Vespa* is for recruitment by giant hornet workers of nestmates during group attacks on colonies of other social insects. Both *V. mandarinia* and *V. soror* workers rub their gasters on the surfaces of prey nests during the early stages of an attack [11,12,13,32]. Field observations suggest that the Richards and other gastral glands may also be involved in this behavior [32]. The most well-supported explanation for the substantial size of the giant hornets’ sternal glands is their use for this purpose. However, the closely related species *V. tropica* and *V. ducalis* have similarly sized gland features. *V. tropica*, while often considered a solitary hunter [9,79], has also been reported to attack prey wasp colonies in groups of nestmates. In Japan, Sakagami and Fukushima [86] observed that *V. tropica* workers return to prey nests with one or two nestmates after an initial attack by one hornet. Seeley et al. [87] described a half-hour group attack in Thailand by three *V. tropica* workers that resulted in the forced abandonment of an *A. florea* nest, which the hornets subsequently plundered. In Vietnam, we also observed the assembly of *V. tropica* nestmates at the nest of honey bee prey; in one instance, three *V. tropica* workers performed oral trophallaxis on an *A. cerana* bee hive. Polistine wasps are considered the species’ main prey [9,86], which is the case for *V. ducalis* too [79]. Presumably, hunting in groups may improve the success of attacks on other colonies of social insect prey, as it does for giant hornets. Observations such as these have prompted speculation that *V. tropica* workers use pheromone communication for nestmate recruitment in the field [86], which would be an intriguing explanation for their well-developed sternal glands.

Another scenario in which pheromone-mediated recruitment by hornet workers could be important is during nest relocation. Of the species that we studied, *V. crabro*, *V. simillima*, and *V. velutina* relocate nests to secondary locations when they outgrow their original nest locations, at high rates of up to 56%, 90%, and 70% of colonies, respectively [1,2,6,9,62,63,64,65,66,67]. Scout workers settle at candidate nest sites and are visited by other workers; they accumulate over days at a preferred site and are eventually joined by the queen. The signals that coordinate this process are unknown [6], but it is hypothesized that chemical communication may facilitate these moves, which can involve travel up to 200 m from the original nest [1]. In some polistines, secretions from the Richards gland are used to guide swarm movement [46,47], although Richards glands are not found in all swarming species [26]. If sternal glands play a signaling role during nest relocation in *V. simillima*, *V. velutina*, or *V. crabro*, it is not manifested as relatively larger Richards glands compared to hornet species that do not construct secondary nests. Giant hornets have the largest Richards glands (scaled for size), but they excavate their nest cavities to accommodate growth rather than undergoing nest relocation. We are unable to make inferences about what drives selection for the Richards gland in *Vespa* workers, primarily because there is so little information available about how and when they use this gland. To date, evidence is limited to the attraction of *V. velutina* males to Richards gland secretions from gynes [48]. This mystery is not restricted to this genus only. Decades since it was first described by Richards [49], a broad purpose for this eponymous gland remains elusive, despite it being as large or many times larger than the van der Vecht gland in all hornet workers that were examined. The Richards gland must have an important function, but we do not yet understand how hornets use it.

## 5. Conclusions

While workers of all *Vespa* species that we examined had van der Vecht and Richards glands in similar configurations, these glands varied in the extent to which they were expressed externally. These differences persisted even when interspecific size differences were considered. Nevertheless, it was difficult to tie differences in morphology to the selective pressures that various subgroups of these species face, aside from hefty gland features that support group-hunting in the giant hornets (and possibly close relatives in their clade). For the most part, this effort was stymied by a lack of information about the use of sternal glands by hornet workers, a problem that extends to exocrine gland function for social wasps generally [43]. Moreover, many authors have highlighted how difficult it is to observe the behavior of defensive hornets within their well-fortified nests [1,6,42]. Future fieldwork that can overcome these barriers and focus on the social contexts that we have outlined may illuminate the selective pressures that have shaped sternal gland morphology in the hornets. This research would be complemented by studies of the chemistry and internal fine structure of the pheromone-producing glands within *Vespa* (e.g., [54]).

## Figures and Tables

**Table 1:** Sources of worker specimens for nine *Vespa* species, with available details about their locations, geographical coordinates, and collection dates (month/year if known).

Species	n	Location	Coordinates	Date	Source
*V. soror* du Buysson, 1905	3	Da Chong, Ba Vi district, Hanoi Prov., Vietnam	21.118° N, 105.335° E	September 2013	G.W. Otis, pers. coll.
	2	Muong Leo, Sop Cop district, Son La Prov., Vietnam	20.836° N, 103.308° E	October 2020	L.T.P. Nguyen, pers. coll.
*V. mandarinia* Smith, 1852	1	Tamugawa-gakuen, Machida City, Tokyo, Japan	35.56° N, 139.46° E	September 2019	M. Ono, Tamagawa Univ.
	1	Lianhuachi Nursery, Nantou County, Taiwan	23.922° N, 120.886° E	August 2015	J.-T. Chao, Taiwan Forestry Res. Inst.
*V. tropica* (Linnaeus, 1758)	1	Da Chong, Ba Vi district, Hanoi Prov., Vietnam	21.118° N, 105.335° E	September 2013	G.W. Otis, pers. coll.
*V. ducalis* Smith 1852	2	Ngoc Dong, Yen Lap district, Phu Tho Prov., Vietnam	21.242° N, 105.150° E	August 2013	G.W. Otis, pers. coll.
*V. crabro* Linnaeus, 1758	1	Plougras, Brittany, France	49.498° N, 3.546° E	September 2018	S. Paiero, Univ. Guelph Insect Coll.
	3	Leuven, Belgium	50.876° N, 4.701° E	September 2020	J. Billen, pers. coll.
	1	Oakville, Ontario, Canada	43.466° N, 79.785°W	September 2013	S. Paiero, Univ. Guelph Insect Coll.
*V. affinis* (Linnaeus, 1764)	1	Da Chong, Ba Vi district, Hanoi Prov., Vietnam	21.118° N, 105.335° E	September 2013	G.W. Otis, pers. coll.
*V. simillima* Smith, 1868	1	Tsukuba Bot. Garden, Akakubo, Tsukuba, Ibaraki Pref., Japan	36.103° N, 140.113° E	July 2020	S. Nomura, Nat. Mus. Nature and Science
*V. velutina* Lepeletier, 1836	1	Taipei Feitsui Reservoir, Taiwan	24.905° N, 121.562° E	June 2020	J.-T. Chao, Taiwan Forestry Res. Inst.
	1	Son Tho, Vu Quang district, Ha Tinh Prov., Vietnam	18.414° N, 105.443° E	May 2012	G.W. Otis, pers. coll.
	2	Sergeac, Aquitaine Region, south France	45.0° N, 1.1° E	2010	A. Perrard, Inst. Ecol. Envir. Sci., Univ. Paris
*V. analis* Fabricius, 1775	1	Tsukuba Bot. Garden, Akakubo, Tsukuba, Ibaraki Pref., Japan	36.103° N, 140.113° E	July 2020	S. Nomura, Nat. Mus. Nature and Science

**Table 2:** Size of features associated with the van der Vecht gland for nine *Vespa* species. Focal species are presented in the order in which they appear in the phylogeny depicted in Figure 1. Pore number includes both lateral clusters. Total area of gland-associated structures includes pore openings and the hyaline region containing the sternal brush. Sternite area extends from its anterior margin to the posterior margin of the smooth cuticle; setal length is given as a percentage of the distance at the midline between the aforementioned cuticular margins (see Figure S1). All estimates were pooled across images of specimens within species. Different letters indicate significant differences among species in pore diameter. Pore number for each species was rounded to the closest hundred.

Species	Number of Pores	Pore Area (mm^2^)	Hyaline Area (mm^2^)	Total Area (mm^2^)	Sternite Area (mm^2^)	Pore Diameter (μm ± SD)	% Length of Setae Relative to Sternite
*V. soror*	4700	0.76	2.85	3.61	8.14	3.8 ± 0.5 a	69.3
*V. mandarinia*	5300	0.90	3.39	4.29	9.91	4.1 ± 0.8 a	62.9
*V. tropica*	5900	1.08	1.53	2.61	6.62	4.4 ± 0.7 b	58.7
*V. ducalis*	3700	0.93	1.77	2.70	6.41	5.0 ± 0.7 c	58.7
*V. crabro*	3200	0.20	0.87	1.07	3.67	2.9 ± 0.4 d	56.2
*V. affinis*	2100	0.24	0.52	0.76	3.85	3.0 ± 0.5 d	29.4
*V. simillima*	1200	0.23	0.21	0.44	2.36	2.8 ± 0.6 d	34.3
*V. velutina*	1500	0.18	0.29	0.47	2.51	2.8 ± 0.4 d	30.9
*V. analis*	1500	0.22	1.96	2.18	5.30	2.9 ± 0.5 d	38.9

**Table 3:** Size of features associated with the Richards gland for nine *Vespa* species. Species are presented in the order in which they appear in the phylogeny depicted in Figure 1. Sternite area extends from the anterior cuticle margin to posterior margin where the smooth cuticle ends (see Figure S1). All estimates were pooled across images of specimens within species. Different letters indicate significant differences among species in pore diameter. Pore number for each species was rounded to the closest hundred.

Species	Number of Pores	Pore Area (mm^2^)	Sternite Area (mm^2^)	Pore Diameter (μm ± SD)
*V. soror*	15,500	1.92	10.75	5.0 ± 0.5 a
*V. mandarinia*	21,500	2.76	12.65	5.0 ± 0.6 a
*V. tropica*	6500	1.18	7.80	4.5 ± 0.6 b
*V. ducalis*	8200	1.49	9.04	4.9 ± 0.6 a
*V. crabro*	3400	0.72	6.22	2.8 ± 0.6 e
*V. affinis*	1600	0.50	4.80	3.1 ± 0.4 d
*V. simillima*	3000	0.41	3.69	2.9 ± 0.5 e
*V. velutina*	2400	0.44	3.65	3.7 ± 0.8 d
*V. analis*	6100	0.75	6.87	3.8 ± 0.4 c

## Data Availability

Data and statistical codes are provided as Supplementary Materials.

## Supplementary Materials

The following supporting information can be downloaded at: https://www.mdpi.com/article/10.3390/biology11020245/s1, Figure S1: Measurements taken from specimens, with images of *V. velutina* as an example; Table S1: Size of features associated with the van der Vecht and Richards glands for workers of the same species, but from different geographical locations; Excel File S1: Data and code from the study.
