# Supplementary material for: Comparison of the External Morphology of the Sternal Glands for Hornets in the Genus Vespa
Source: Biology (Basel). 2022 Feb 5;11(2):245. doi: 10.3390/biology11020245 (PMC8868583; doi:10.3390/biology11020245)
Supplement: Supplementary file 1 [file biology-11-00245-s001.zip › Revised Supplementary Materials, Mattila et al..pdf]

Table S1. Size of features associated with the van der Vecht and Richards glands for workers of the same species, but from different geographical locations. Sternite area extends from the anterior cuticle margin to posterior margin where the smooth cuticle ends (see Figure S1). For the van der Vecht gland, total area of gland structures includes pore openings and hyaline region containing the sternal brush. Values are for single specimens, except for *V. velutina* from France for which averages for two specimens are provided. Pore number per species was rounded to the closest hundred.

Species	Collection location	Number of pores	Sternite area (mm^2)	Pore area (mm^2)	Hyaline area (mm^2)	Total area (mm^2)
		van der Vecht gland				
V. mandarinia	Japan	3900	10.71	0.87	3.65	4.52
	Taiwan	6600	9.10	0.94	3.13	4.07
V. crabro	France	3600	4.06	0.24	0.85	1.09
	Belgium	2800	2.77	0.21	0.72	0.93
	Canada	3000	4.17	0.14	1.04	1.18
V. velutina	Taiwan	1800	2.58	0.21	0.33	0.54
	Vietnam	1600	2.74	0.19	0.34	0.53
	France	900	2.20	0.15	0.21	0.36
		Richards gland				
V. mandarinia	Japan	22400	13.15	2.85		
	Taiwan	20500	12.14	2.65		
V. crabro	France	3600	6.16	0.73		
	Belgium	3200	5.90	0.65		
	Canada	3400	6.59	0.77		
V. velutina	Taiwan	3400	3.78	0.53		
	Vietnam	1800	3.97	0.44		
	France	2000	3.20	0.33		

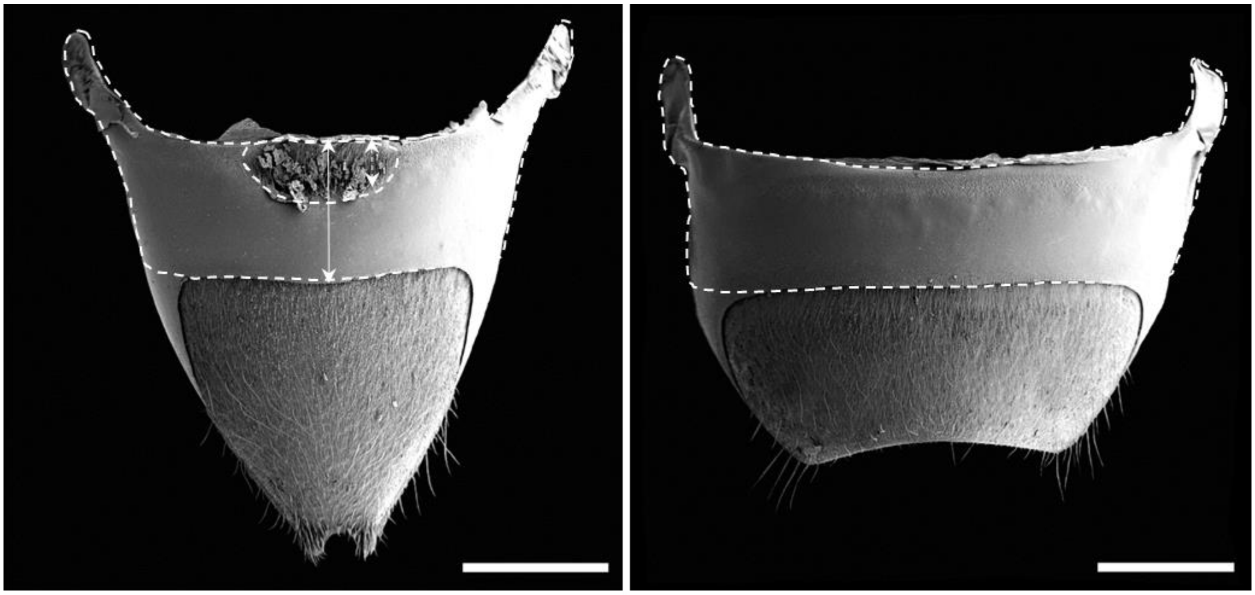

(a) (b)

Figure S1. Measurements taken from specimens, with images of *V. velutina* as an example. Anterior is at the top. The dashed outline indicates the anterior portion of the sternite for which area was measured for each specimen's sternites. (a) Sixth metasomal sternite with the van der Vecht gland. The solid arrow indicates the length of the sternal midline that was measured; the dashed arrow is an example of setal length that was measured in quadrants across the sternal brush. The interior dashed line indicates the hyaline region with the sternal brush. (b) Fifth metasomal sternite with the Richards gland. Bars indicate 1 mm for scale.
